# Cancer of the Bones

**Published:** 1847-06

**Authors:** 


*Cancer of the Bones.*—A late number of the "Gazette des Hopitaux," contains a lecture by Professor Roux, on fungous tumors of the bones. The following has been the issue of the case which gave occasion to the lecture:—The Professor performed ligature of the femoral artery on the 18th of November; sixteen days after, on the the 5th of December, the ligature fell, and an extremely considerable hæmorrhage occurred. Compression of the vessel was instituted, and Professor Roux was sent for. Another ligature was placed one inch higher up on the femoral artery; but, a large vein having been wounded during the operation, phlebitis set in, and the patient died on the 11th of December. On dissection the spleen and liver were found occupied by metastatic abscesses; the right knee was filled with pus, and the tibia was the seat of an evident encephaloid degeneration; but the cartilages of the joint were perfectly healthy.

M. Nelaton distinguishes four forms of cancer in the bones:—1. In the first nodi of cancerous matter, replace the bony tissue in the spongy and in the compact textures, and in the immediate vicinity the bone seems to have remained perfectly healthy. When the disease occupies the diaphysis of the bone it is not uncommon to see a fungous excrescence obliterate the medullary canal, and rise within its cavity far higher than the outward aspect of the disease would lead the observer to suppose; an important remark, which must not be lost sight of when amputation is contemplated. 2. In the second form, generally denominated osteo-sarcoma, the tumor is larger, and is formed by the deposition of cancerous matter in the enlarged cells of the bone. 3. In a third variety, a cancerous growth arises within a bone, and gradually dilates it, being at last surrounded by a very thin osseous shell; this is spina ventosa. 4. The tumor is attached to the bone, but covered by the periosteum, the substance of the bone is, however, diseased, and numerous projections as thin as hairs unite the tumor with the osseous texture beneath.—In all four the swelling increases gradually, becomes softened, ulcerated, returns after amputation, and finally brings on the cancerous cachexy. The cartilages of the joints are never invaded. These cancerous affections are frequently the seat of pulsations which resemble those of aneurism; it is not impossible, however, to find a difference between the pulsations of both orders of diseases, by which they may be readily distinguished. In aneurism, the pulsations exist from the very beginning of the malady; in cancer, on the contrary, they come on only when the swelling has become extremely vascular, *i. e.*, at an advanced period of the existence.

